# The clinical effects of laparoscopic gastrojejunostomy versus nasointestinal tube insertion with subsequent chemotherapy for uncurable gastric cancer patients with outlet obstruction

**DOI:** 10.3389/fonc.2026.1833761

**Published:** 2026-05-21

**Authors:** Zhaojie Ruan, Changshun Yang, Shengtao Lin, Weihua Li

**Affiliations:** 1 Ningde Clinical Medical College of Fujian Medical University, Ningde, Fujian, China; 2 Department of Gastrointestinal Surgery, Ningde Municipal Hospital, Ningde, Fujian, China; 3 Department of Gastrointestinal Surgery, Fujian Provincial Hospital, Fuzhou University Affiliated Provincial Hospital, School of Medicine, Fuzhou University, Fuzhou, Fujian, China

**Keywords:** LGJ, NIT, outcome, outlet obstruction, uncurable gastric cancer

## Abstract

**Objective:**

To compare the clinical outcomes of laparoscopic gastrojejunostomy (LGJ) versus nasointestinal tube (NIT) insertion, both combined with chemotherapy, in patients with advanced gastric cancer and gastric outlet obstruction (GOO), and to provide evidence for optimal management strategies.

**Methods:**

This retrospective study included 63 patients with incurable advanced gastric cancer and GOO treated at Fujian Provincial Hospital, between January 2019 and December 2022. Patients were assigned to either the LGJ group (n=33) or the NIT group (n=30). Clinical baseline data, short-term complications, quality of life (QOL), nutritional and inflammatory markers, chemotherapy response, and conversion surgery outcomes were compared. Prognostic factors for overall survival (OS) were evaluated using univariate and multivariate Cox regression analyses.

**Results:**

Baseline characteristics were comparable between groups. No severe complications occurred in the LGJ group. Post-obstruction, LGJ significantly improved GOOSS scores, with 90.9% of patients resuming full oral intake, compared to 30% unable to eat in the NIT group. The LGJ group had longer hospital stays and delayed chemotherapy initiation but showed lower rates of elevated NLR and CEA (P<0.05). QOL declined more frequently in the NIT group (P<0.05). No significant differences were observed in chemotherapy response, conversion surgery rates (30.3% vs 23.3%), or pathological outcomes. Median OS was 18.3 months in the LGJ group vs 14.3 months in the NIT group (P = 0.453). Borrmann classification and conversion surgery were independent prognostic factors for OS.

**Conclusion:**

LGJ offers superior improvement in nutritional status and QOL compared to NIT, with comparable survival outcomes. Borrmann type and conversion surgery are key predictors of long-term prognosis in this patient population.

## 1 Introduction

Gastric cancer, as a lethal malignancy that poses a serious threat to human health (1), is often diagnosed at an advanced or late stage at initial presentation in China (2). In addition, gastric cancer commonly exhibits pronounced tumor heterogeneity and aggressive biological behavior, leading to generally poor prognosis (3, 4). To date, radical surgery remains the only curative approach for gastric cancer. However, due to its insidious onset and the population’s fear of invasive procedures such as gastroscopy, a considerable number of patients have already lost the opportunity for curative surgery at the time of diagnosis (5). In recent years, along with advancements in basic research and several high-quality clinical studies, non-surgical treatments for gastric cancer have also made progress, including the optimization of chemotherapy regimens and the emergence and gradual application of targeted therapy and immunotherapy. Significant changes have also occurred in treatment strategies and concepts: neoadjuvant therapy and conversion therapy have, to varying degrees, improved resectability rates and clinical outcomes for patients (4, 6–8).However, in distal gastric cancer, gastric outlet obstruction (GOO) represents one of the most devastating pathological states in advanced disease.

Gastrojejunostomy has long been an effective palliative surgical approach for managing GOO in advanced gastric cancer (9). With the rise and maturation of laparoscopy, laparoscopic gastrojejunostomy (LGJ) has rapidly gained popularity and gradually become mainstream due to its minimally invasive nature and thorough intra-abdominal evaluation capabilities (10, 11). Following gastrojejunostomy, the digestive tract is reconnected, allowing patients to resume oral intake, thereby significantly improving their nutritional status and quality of life, and helping them achieve better physical and psychological preparation for subsequent antitumor treatment (12).Other commonly used methods to relieve or alleviate GOO include endoscopic placement of self-expanding metal stents (SEMS) (13, 14), nasointestinal tube (NIT) placement (15), or jejunostomy (16), and in emergencies such as bleeding or perforation, palliative resection may be performed out of necessity (17).

Therefore, this study aims to compare the short-term outcomes (postoperative complications, nutritional indices, inflammatory and tumor markers) and long-term outcomes (overall survival, OS) of LGJ versus NIT combined with chemotherapy in the treatment of patients with advanced incurable GOO gastric cancer. We further aim to identify factors associated with overall survival to provide a reference for treatment selection in such patients.

## 2 Methods

### 2.1 Patients

This study included 63 patients with incurable advanced gastric cancer accompanied by gastric outlet obstruction (GOO) who were treated at the Department of Gastrointestinal Surgery of Fujian Provincial Hospital from January 1, 2019 to December 31, 2022. The patients were divided into two groups: the laparoscopic gastrojejunostomy (LGJ) group (n=33) and the nasointestinal tube (NIT) placement group (n=30). Inclusion criteria: (1) Age between 18 and 80 years; (2) Pathological confirmation of unresectable gastric adenocarcinoma via gastroscopic biopsy and CT imaging, with incurable factors such as invasion of adjacent organs, liver metastasis, peritoneal metastasis, or distant lymph node metastasis; (3) Evidence of gastric retention and malignant gastric outlet narrowing under gastroscopy, along with varying degrees of GOO symptoms; (4) Underwent LGJ or NIT insertion for relief of GOO before systemic chemotherapy and subsequently received at least two cycles of S-1 plus oxaliplatin (SOX)-based conversion chemotherapy;(5) No severe cardiac or pulmonary dysfunction or other severe comorbidities; (6) Eastern Cooperative Oncology Group (ECOG) performance status score 0–2; (7) No history of systemic chemotherapy, targeted therapy, immunotherapy, or radiotherapy before the diagnosis of GOO and the initial obstruction-relieving intervention. Exclusion criteria: (1) Uncontrollable active bleeding or perforation requiring emergency surgery; (2) Malignant gastric outlet narrowing not caused by gastric cancer; (3) Presence of other malignancies. All included patients presented with GOO at the time of initial diagnosis and had not received systemic antitumor therapy before the obstruction-relieving intervention. No patient developed GOO during treatment for previously diagnosed gastric cancer.

This study was approved by the Ethics Committee of Fujian Provincial Hospital and it adhered to the principles of the Helsinki Declaration. This study obtained informed consent from all patients and their families.

### 2.2 Surgical/procedural and chemotherapy protocols

(1) Laparoscopic Gastrojejunostomy: Under general anesthesia with endotracheal intubation, patients were placed in the scissors position. After routine disinfection and draping, catheterization was performed. A pneumoperitoneum was established via a subumbilical puncture needle with CO^2^ insufflation maintained at 13 mmHg. Ports were placed: subumbilical for observation, left upper abdomen as main operating port, and bilateral middle abdomen as auxiliary ports. Laparoscopic exploration was conducted to examine for ascites, pelvic nodules, omental or hepatic metastases, and tumor invasion in the antrum, with images or videos recorded. The stomach was gently lifted with non-traumatic forceps, and the greater omentum was dissected along the greater curvature with an ultrasonic scalpel. A hole was made at the greater curvature for anastomosis. A side-to-side antiperistaltic gastrojejunostomy was created using a linear stapler between the stomach and the proximal jejunum 30–35 cm distal to the Treitz ligament. A second side-to-side jejunojejunostomy (Braun anastomosis) was performed between the jejunum 15 cm distal to the Treitz ligament and 40 cm distal to the gastrojejunostomy site.
(2) Endoscopic Nasointestinal Tube Placement: Patients with gastric retention underwent decompression and lavage two days prior. Placement was conducted under endoscopy by experienced endoscopists under sedation as needed. The degree of malignant gastric outlet narrowing was evaluated and documented. A nasointestinal tube or dual-lumen tube (for decompression and enteral nutrition) was inserted into the descending duodenum or further, and confirmed with an abdominal X-ray.
(3) Chemotherapy Protocol: All patients received S-1 and oxaliplatin (SOX) chemotherapy (4), including oxaliplatin intravenous infusion (85–130 mg/m² on Day 1) and S-1 (80–120 mg/m², twice daily for 14 days), administered orally or via feeding tube, repeated every 3 weeks. All patients received SOX-based conversion chemotherapy before surgery. No targeted therapy or immunotherapy was incorporated into the preoperative conversion regimen during the study period. The chronological treatment sequence was diagnosis of advanced gastric cancer with GOO, obstruction-relieving intervention with either LGJ or NIT, recovery or establishment of an enteral feeding route, and subsequent initiation of SOX chemotherapy. In patients unable to tolerate oral intake, S-1 was administered through the nasointestinal feeding tube when feasible.

Treatment allocation and decision-making. Both LGJ and NIT were considered technically available for all enrolled patients after evaluation by the treating team, including assessment of ECOG performance status, cardiopulmonary function, anesthetic risk, severity of obstruction, and expected tolerance to chemotherapy. Patients who required emergency surgery for uncontrolled bleeding or perforation were excluded. After multidisciplinary and surgeon-led evaluation, physicians explained the potential advantages and disadvantages of both approaches to patients and their families. LGJ was generally discussed as an option providing more durable relief of obstruction and better restoration of oral intake, whereas NIT was discussed as a less invasive approach that could allow earlier initiation of systemic chemotherapy. The final treatment choice was made jointly by patients, families, and physicians according to clinical suitability, patient preference, perceived surgical risk, willingness to undergo an operation, expected time to chemotherapy, and economic considerations. No patient was assigned to either group by randomization. Therefore, although baseline clinicopathological characteristics were comparable between groups, residual selection bias related to unmeasured patient or physician factors could not be completely excluded.

### 2.3 Nutritional assessment and support

The treatment strategy for each patient was determined by a multidisciplinary team consisting of oncology, nutrition, and surgery specialists. Nutritional assessment and nutritional support were part of the standard management protocol during hospitalization. Nutritional support was individualized according to the severity of gastric outlet obstruction, oral intake ability, gastrointestinal tolerance, body weight, and the planned chemotherapy schedule.

In the LGJ group, oral intake was gradually resumed after recovery of gastrointestinal function. On postoperative day 1, patients were encouraged to drink 500–1000 ml of clear fluid, and the amount of oral intake was gradually increased according to individual tolerance. Parenteral nutrition was discontinued when oral intake reached approximately 2000–2500 ml/day. Additional enteral or parenteral nutritional support was provided when spontaneous oral intake was considered insufficient.

In the NIT group, enteral nutrition was delivered through the nasointestinal tube during hospitalization. The enteral nutrition formula used in our center contained protein 4.0 g, fat 3.0 g, and carbohydrate 12.1 g per unit, with a caloric density of 1.0 kcal/ml, and was administered at approximately 40 °C. The target caloric and protein intake were generally set at 25–30 kcal/kg/day and 1–2 g/kg/day, respectively. When enteral nutrition alone was insufficient or temporarily not tolerated, supplemental parenteral nutrition was administered according to the patient’s clinical condition.

Because this was a retrospective study, detailed daily caloric and protein intake records were not uniformly available for all patients. Therefore, nutritional outcomes were mainly evaluated using clinical and laboratory indicators, including Gastric outlet obstruction scoring system (GOOSS), serum albumin, PNI, Nutritional Risk Screening 2002(NRS2002), and Patient-Generated Subjective Global Assessment (PG-SGA).

### 2.4 Observational indicators

(1) Clinical and Pathological Baseline Characteristics: Age, sex, BMI, ECOG score, GOOSS score, QOL score, NRS2002 score, PG-SGA score, Prognostic Nutritional Index (PNI), Neutrophil-to-Lymphocyte Ratio (NLR), Platelet-to-Lymphocyte Ratio (PLR), CEA, CA19-9, clinical TN staging (cT, cN), and reasons for unresectability were recorded. Gastric outlet obstruction scoring system (GOOSS) is defined as follows: 0, no oral intake; 1, liquid only; 2, soft food; and 3, low-residue or full diet (10). Quality of life (QOL) was assessed using the Spitzer QOL-Index, a concise clinician-rated instrument covering five domains: activity, daily living, health, support, and outlook. Each domain is scored from 0 to 2, with 0 indicating the poorest status and 2 indicating the best status; the total score therefore ranges from 0 to 10, with higher scores reflecting better QOL (11). Nutritional status was evaluated using the Nutritional Risk Screening 2002 (NRS2002), the Patient-Generated Subjective Global Assessment (PG-SGA), serum albumin, and PNI. NRS2002 was used to screen nutritional risk, with a score ≥3 indicating nutritional risk. PG-SGA was used to assess cancer-related malnutrition, with higher scores indicating greater nutritional impairment (14).
(2) Clinical Outcomes: Short-term outcomes included postoperative complications (bleeding, perforation, leakage, recurrent obstruction), hospital stay, GOOSS improvement, and time to first chemotherapy. Long-term outcomes included tumor response to chemotherapy (CR, PR, SD, PD) and resection rate after conversion therapy. CR: Complete Response; PR: Partial Response; SD: Stable Disease; PD: Progressive Disease; ORR: Objective Response Rate.
(3) Pathological Data of Patients Undergoing Surgery Post-Conversion: Resection margin status (R0 = negative; R1 = microscopic positive; R2 = macroscopic positive), Tumor Regression Grade (TRG0–3), and postoperative pathological stage (pT, pN).

TRG was evaluated according to the Becker grading system: TRG0, no residual tumor cells; TRG1, <10% residual tumor cells; TRG2, 10-50% residual tumor cells; and TRG3, >50% residual tumor cells (7). Macroscopic tumor type was classified according to the Borrmann classification, including type I polypoid tumors, type II ulcerated tumors with clear margins, type III ulcerated infiltrative tumors, and type IV diffusely infiltrative tumors (5).

(4) Follow-up

SOX chemotherapy was continued for 6–8 cycles or until unacceptable toxicity, treatment refusal, or evidence of resectability. Routine blood tests, biochemistry, and tumor markers were reviewed before each cycle; enhanced abdominal CT was repeated every two cycles. Within one month, inflammatory and nutritional markers were assessed. After approximately three months, GOOSS, QOL, NRS2002, PG-SGA, CEA, and CA19–9 were re-evaluated. In our center, resectability after chemotherapy was determined through a multidisciplinary team (MDT) discussion involving surgical oncology, medical oncology, radiology, and gastroenterology. The MDT primarily evaluated contrast-enhanced CT findings, and tumors achieving a radiologic complete response (CR) or partial response (PR) were generally considered resectable. And it was the key and consistent threshold for initiating MDT resectability assessment throughout the study period. Response to chemotherapy was classified according to the Response Evaluation Criteria in Solid Tumors guidelines (version 1.0) Additional clinical factors, including performance status, nutritional condition, and absence of prohibitive comorbidities, were also incorporated into the decision-making process. Postoperative chemotherapy was guided by clinical protocols.

### 2.5 Statistical analysis

Quantitative variables were analyzed using the independent t-test or Wilcoxon rank-sum test, depending on distribution and variance. Categorical variables were analyzed using the Chi-square test or Fisher’s exact test. A p-value < 0.05 was considered statistically significant. Overall survival (OS) was defined as the time from initiation of chemotherapy to death or last follow-up. OS was analyzed using the Kaplan-Meier method and log-rank test. Univariate and multivariate analyses were performed using Cox regression. All analyses were conducted with SPSS (IBM SPSS Statistics, Version 22.0).

## 3 Results

### 3.1 Baseline data

This retrospective study included a total of 63 patients with incurable advanced gastric cancer complicated by gastric outlet obstruction (GOO). Patients were divided into two groups: the laparoscopic gastrojejunostomy (LGJ) group (n=33) and the endoscopic nasointestinal tube (NIT) group (n=30). Baseline clinical data included age, sex, body mass index (BMI), ECOG/PS score, clinical T stage, clinically positive lymph nodes, serum CEA level, Borrmann classification, serum inflammatory markers (PNI, PLR, NLR), gastric outlet obstruction score (GOOSS), quality of life assessment (QOL score), and causes of unresectability (such as invasion of surrounding tissues, peritoneal metastasis, liver metastasis, and distant lymph node metastasis), as shown in **Table 1**. In the LGJ group, 8 patients presented with peritoneal metastasis concurrently with distant lymph node metastasis, and 6 patients had hepatic metastasis accompanied by distant lymph node involvement; the remaining cases exhibited a single non-curable factor. In the NIT group, 4 patients showed both peritoneal and distant lymph node metastasis, while 3 patients had hepatic metastasis together with distant lymph node involvement, with all other patients presenting a single non-curable factor. Statistical analysis showed that the two groups were matched in demographic characteristics such as age, sex, and BMI, indicating good comparability. There were no statistically significant differences between the two groups in terms of nutritional status, quality of life, inflammatory status, tumor marker positivity, tumor stage distribution, or causes of unresectability.

**Table 1:** Baseline patients characteristics.

Characteristics	LGJ(n=33)	NIT(n=30)	*P value*
Age	61.79±12.51	62.10±11.90	0.920
Sex(Male/Female)	21/12(63.6/36.4)	20/10(66.7/33.3)	0.801
BMI(kg/m^2^)	21.69±2.52	21.53±2.16	0.783
ECOG(0/1/2)	2/22/9(6.1/66.7/27.3)	3/21/6(10.0/70.0/20.0)	0.754
cT(T3/T4a/T4b)	4/27/2(12.1/81.8/6.1)	3/24/3(10.0/80.0/10.0)	0.899
cN(+)	33/33(100)	30/30(100)	–
CEA(ng/mL)			0.387
< 5	19(57.6)	14(46.7)	
≥ 5	14(42.4)	16(53.3)	
Bormann			0.424
III	26(78.8)	21(70.0)	
IV	7(21.2)	9(30.0)	
PNI			0.583
< 45	22(66.7)	18(60.0)	
≥ 45	11(33.3)	12(40.0)	
NLR			0.108
< 2.5	8(24.2)	13(43.3)	
≥ 2.5	25(75.8)	17(56.7)	
PLR			0.182
< 162	10(30.3)	14(46.7)	
≥ 162	23(69.7)	16(53.3)	
Albumin(g/L)	36(27-42)	35(27-41)	0.278
GOOSS			0.516
0	17(51.5)	13(47.6)	
1	16(48.5)	17(52.4)	
QOL	5.88±1.34	5.87±1.14	0.969
Non-curable factor			
Infiltration to adjacent organs	5(15.2)	3(10.0)	0.710
Peritoneal metastasis	18(54.5)	14(46.7)	0.532
Hepatic metastasis	6(18.2)	7(23.3)	0.614
Distant lymph node metastasis	18(54.5)	13(43.3)	0.374

LGJ: Laparoscopic Gastrojejunostomy, NIT: Nasointestinal Tube Insertion, PNI: Prognostic Nutritional Index, BMI: Body mass index, PLR: Platelet to lymphocyte ratio, NLR: Neutrophil to lymphocyte ratio, GOOSS: Gastric outlet obstruction scoring system, PNI: Prognostic Nutritional Index, BMI: Body mass index, PLR: Platelet to lymphocyte ratio, NLR: Neutrophil to lymphocyte ratio, QOL: Quality of life.

### 3.2 Clinical outcomes after interventions

The clinical outcomes of the interventions are summarized in **Table 2**. No serious complications such as bleeding, perforation, or leakage were observed in the LGJ group postoperatively. Following the bypass, the vast majority of LGJ patients resumed a full oral diet (GOOSS score of 3, 90.9%), while none of the patients in the NIT group achieved a GOOSS score of 3. In the NIT group, 30% were unable to eat orally, 56.7% were able to consume liquids, and 13.3% could ta7ke semi-liquid food. Hospital stay and time to initiation of chemotherapy were both longer in the LGJ group compared to the NIT group (7 [5–14] vs 2 [1–5] days; 14 [9–22] vs 8 [6–14] days, both p < 0.05). The proportions of patients with NLR ≥ 2.5 and CEA positivity were lower in the LGJ group than in the NIT group (45.5% vs 73.3% and 51.5% vs 76.7%, both p < 0.05). However, there were no statistically significant differences in the proportions of patients with PNI < 45 or PLR < 162 between the two groups. A higher proportion of patients in the NIT group experienced a decline in QOL (stable/improved vs declined: 22/8 [73.3%/26.7%] vs 31/2 [93.9%/6.1%], p < 0.05). Serum albumin levels were significantly higher in the LGJ group compared to the NIT group, and albumin levels in the LGJ group were also significantly increased after palliative diversion (p < 0.05). There were no significant differences between the groups in chemotherapy response or objective response rate (ORR: 51.5% vs 30%, p > 0.05). The median number of chemotherapy cycles was higher in the LGJ group than in the NIT group [6 (2-10) vs 4 (2-8), P = 0.0428], suggesting that patients in the LGJ group were able to receive more cycles of systemic treatment after relief of obstruction.

**Table 2:** Clinical outcomes of LGJ+chemotherapy versus NIT+chemotherapy.

Clinical outcomes	LGJ(n=33)	NIT(n=30)	* P value*
Postoperative bleeding, perforation and fistula	0(0.0)	–	–
GOOSS			< 0.001
0	0(0.0)	9(30)	
1	0(0.0)	17(56.7)	
2	3(9.1)	4(13.3)	
3	30(90.9)	0(0.0)	
Length of hospital stay	7(5-14)	2(1-5)	< 0.001
The time of the first chemotherapy	14(9-22)	8(6-14)	< 0.001
CEA(ng/mL)			0.038
< 5	16(48.5)	7(23.3)	
≥ 5	17(51.5)	23(76.7)	
PNI			0.236
< 45	17(51.5)	11(36.7)	
≥ 45	16(48.5)	19(63.3)	
NLR			0.025
< 2.5	18(54.5)	8(26.7)	
≥ 2.5	15(45.5)	22(73.3)	
PLR			0.204
< 162	10(30.3)	5(23.8)	
≥ 162	23(69.7)	25(76.2)	
Albumin(g/L)	42(30-61)	34(30-43)	< 0.001
QOL (increase or remain unchanged/decrease)	31/2(93.9/6.1)	22/8(73.3/26.7)	0.038
Chemotherapy cycles	6 (2–10)	4 (2–8)	0.0428
Chemotherapy remission situation			
CR	3(9.1)	2(6.7)	0.363
PR	14(42.4)	7(23.3)	
SD	7(21.2)	13(43.3)	
PD	9(27.3)	8(26.7)	
ORR(%)	51.5	30.0	0.083
Surgical resection rate	30.3(10/33)	23.3(7/30)	0.534

LGJ: Laparoscopic Gastrojejunostomy, NIT: Nasointestinal Tube Insertion, PNI: Prognostic Nutritional Index, BMI: Body mass index, PLR: Platelet to lymphocyte ratio, NLR: Neutrophil to lymphocyte ratio, GOOSS: Gastric outlet obstruction scoring system, PNI: Prognostic Nutritional Index, BMI: Body mass index, PLR: Platelet to lymphocyte ratio, NLR: Neutrophil to lymphocyte ratio, QOL: Quality of life.

A total of 17 patients underwent conversion surgery, including 10 in the LGJ group and 7 in the NIT group. The two groups were generally comparable in age, sex, BMI, ECOG status, clinical T stage, CEA, Borrmann classification, inflammatory and nutritional markers, baseline GOOSS, and QOL score. Among these patients, adjacent organ invasion was present in 7 patients, liver metastasis in 2 patients, and distant lymph node metastasis in 13 patients. No patient with peritoneal metastasis underwent conversion surgery in this cohort. Specifically, in the LGJ group, 5 patients had adjacent organ invasion, 1 had liver metastasis, and 8 had distant lymph node metastasis; in the NIT group, 2 patients had adjacent organ invasion, 1 had liver metastasis, and 5 had distant lymph node metastasis. (**Table 3**)

**Table 3:** Clinical characteristics of patients undergoing conversion surgery.

Characteristics	LGJ(n=10)	NIT(n=7)	*P value*
Age	58.40 ± 14.41	57.86 ± 14.19	0.940
Sex (Male/Female)	8/2 (80.0/20.0)	4/3 (57.1/42.9)	0.593
BMI (kg/m^2^)	22.70 ± 2.49	21.26 ± 2.81	0.298
ECOG (0/1/2)	2/7/1 (20.0/70.0/10.0)	0/5/2 (0.0/71.4/28.6)	0.332
cT (T3/T4a/T4b)	2/6/2 (20.0/60.0/20.0)	0/6/1 (0.0/85.7/14.3)	0.394
CEA (ng/mL, <5/≥ 5)	8/2 (80.0/20.0)	4/3 (57.1/42.9)	0.593
Borrmann classification (III/IV)	9/1 (90.0/10.0)	4/3 (57.1/42.9)	0.250
PNI (<45/≥ 45)	7/3 (70.0/30.0)	3/4 (42.9/57.1)	0.350
NLR (<2.5/≥ 2.5)	3/7 (30.0/70.0)	2/5 (28.6/71.4)	1.000
PLR (<162/≥ 162)	4/6 (40.0/60.0)	1/6 (14.3/85.7)	0.338
Albumin before intervention (g/L)	34 (30–42)	36 (31–41)	0.432
Baseline GOOSS (0/1)	6/4 (60.0/40.0)	5/2 (71.4/28.6)	1.000
Baseline QOL score	6.10 ± 1.20	6.00 ± 0.82	0.841
Non-curable factor			
Infiltration to adjacent organs	5 (50.0)	2 (28.6)	0.622
Peritoneal metastasis	0 (0.0)	0 (0.0)	—
Hepatic metastasis	1 (10.0)	1 (14.3)	1.000
Distant lymph node metastasis	8 (80.0)	5 (71.4)	1.000
Treatment-related variables			
Interval from chemotherapy to surgery (months)	4.5 (3.0–9)	4.0 (3.0–11.0)	0.681
Chemotherapy cycles before surgery	6 (4–10)	6 (4–8)	0.892

LGJ: Laparoscopic Gastrojejunostomy, NIT: Nasointestinal Tube Insertion, PNI: Prognostic Nutritional Index, BMI: Body mass index, PLR: Platelet to lymphocyte ratio, NLR: Neutrophil to lymphocyte ratio, GOOSS: Gastric outlet obstruction scoring system, PNI: Prognostic Nutritional Index, BMI: Body mass index, PLR: Platelet to lymphocyte ratio, NLR: Neutrophil to lymphocyte ratio, QOL: Quality of life.

For patients with liver metastasis who underwent conversion surgery, local treatment such as radiofrequency ablation was performed when metastatic lesions became controllable after chemotherapy. For patients with adjacent organ invasion, en bloc or combined organ resection was performed when R0 resection was considered feasible. For patients with distant lymph node metastasis, conversion surgery was considered when metastatic lymph nodes markedly regressed or disappeared on imaging and no new metastatic lesions were detected. These details have now been added to the Results section to clarify which incurable factors became surgically manageable after chemotherapy.

### 3.3 Pathological outcomes after conversion surgery

In the LGJ group, 10 patients (30.3%) underwent resection following conversion therapy, while 7 patients (23.3%) in the NIT group underwent surgery after conversion therapy. R0 resection was achieved in 8 patients (80.0%) in the LGJ group and 6 patients (85.7%) in the NIT group (**Table 4**). Postoperative pathology confirmed complete pathological response (TRG0) in 3 patients in the LGJ group and 2 patients in the NIT group. There were no statistically significant differences in the distribution of TRG grades between the two groups. Among patients who did not achieve complete response, the pathological T stage was primarily concentrated at T4a (60.0% in LGJ group vs 57.1% in NIT group). Notably, a substantial proportion of patients had negative lymph nodes postoperatively (80.0% in LGJ vs 57.1% in NIT). There were no significant differences between the groups in resection margin status, pathological response grade, or postoperative tumor stage.

**Table 4:** Surgical and pathological outcomes of patients underwent conversion surgery.

Surgical and pathological outcomes	LGJ(n=10)	NIT(n=7)	*P value*
Resection edge			
R0	8(80.0)	6(85.7)	1.000
R1	2(20.0)	1(14.3)	
TRG			
0	3(30.0)	2(28.6)	1.000
1	2(20.0)	1(14.3)	
2	3(30.0)	2(28.6)	
3	2(20.0)	2(28.6)	
Postoperative pathology			
pT			
0	3(30.0)	2(28.6)	0.870
1	0(0.0)	0(0.0)	
2	0(0.0)	1(14.3)	
3	1(10.0)	0(0.0)	
4a	6(60.0)	4(57.1)	
pN			
(-)	8(80.0)	4(57.1)	0.593
(+)	2(20.0)	3(42.9)	

LGJ: Laparoscopic Gastrojejunostomy, NIT: Nasointestinal Tube Insertion, TRG: Tumor Regression Grade.

4. Survival Analysis and Risk Factor Assessment

**Figure 1:**
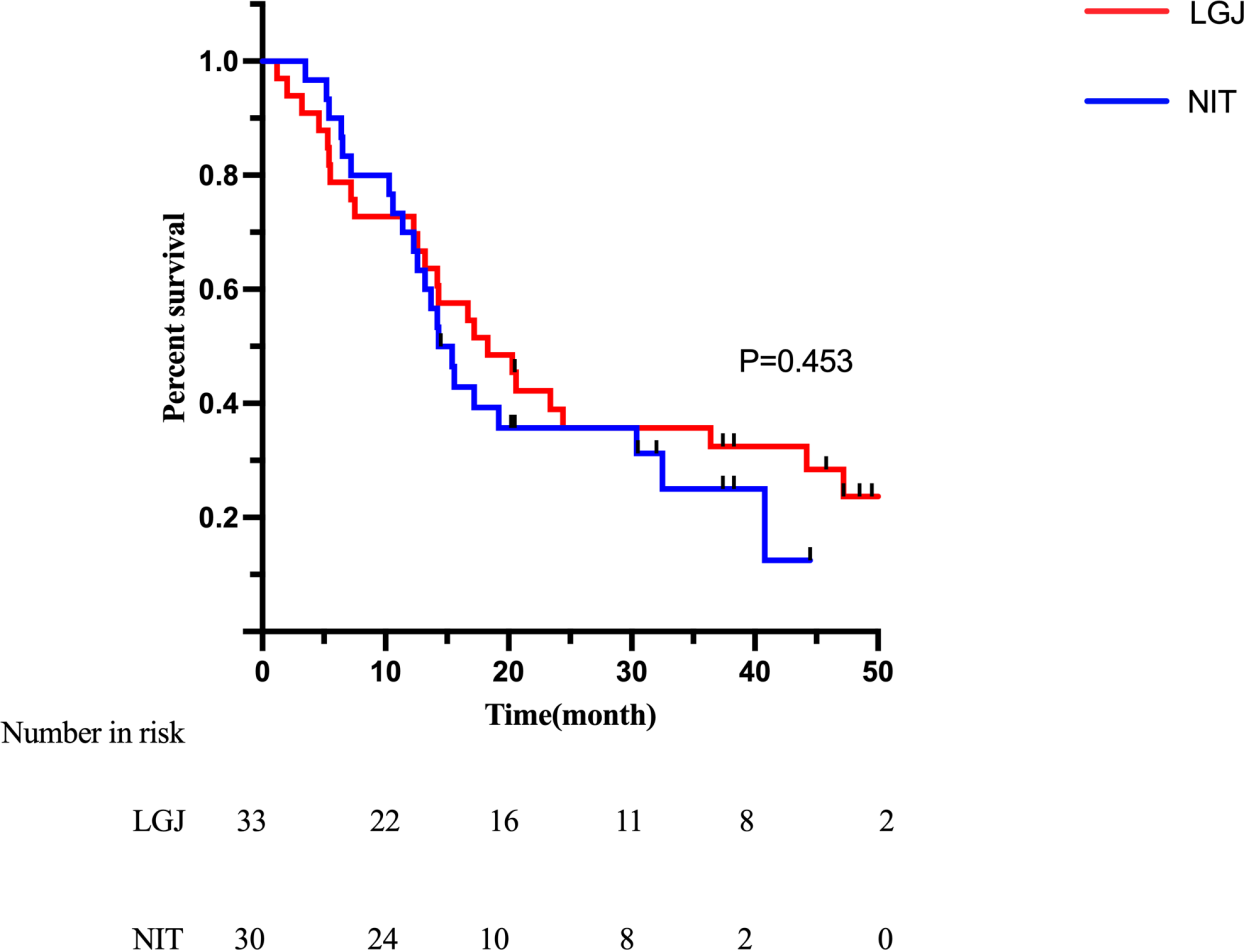
Kaplan-Meier analysis for the overall survival of two groups. LGJ, Laparoscopic Gastrojejunostomy; NIT, Nasointestinal Tube Insertion.

**Figure 2:**
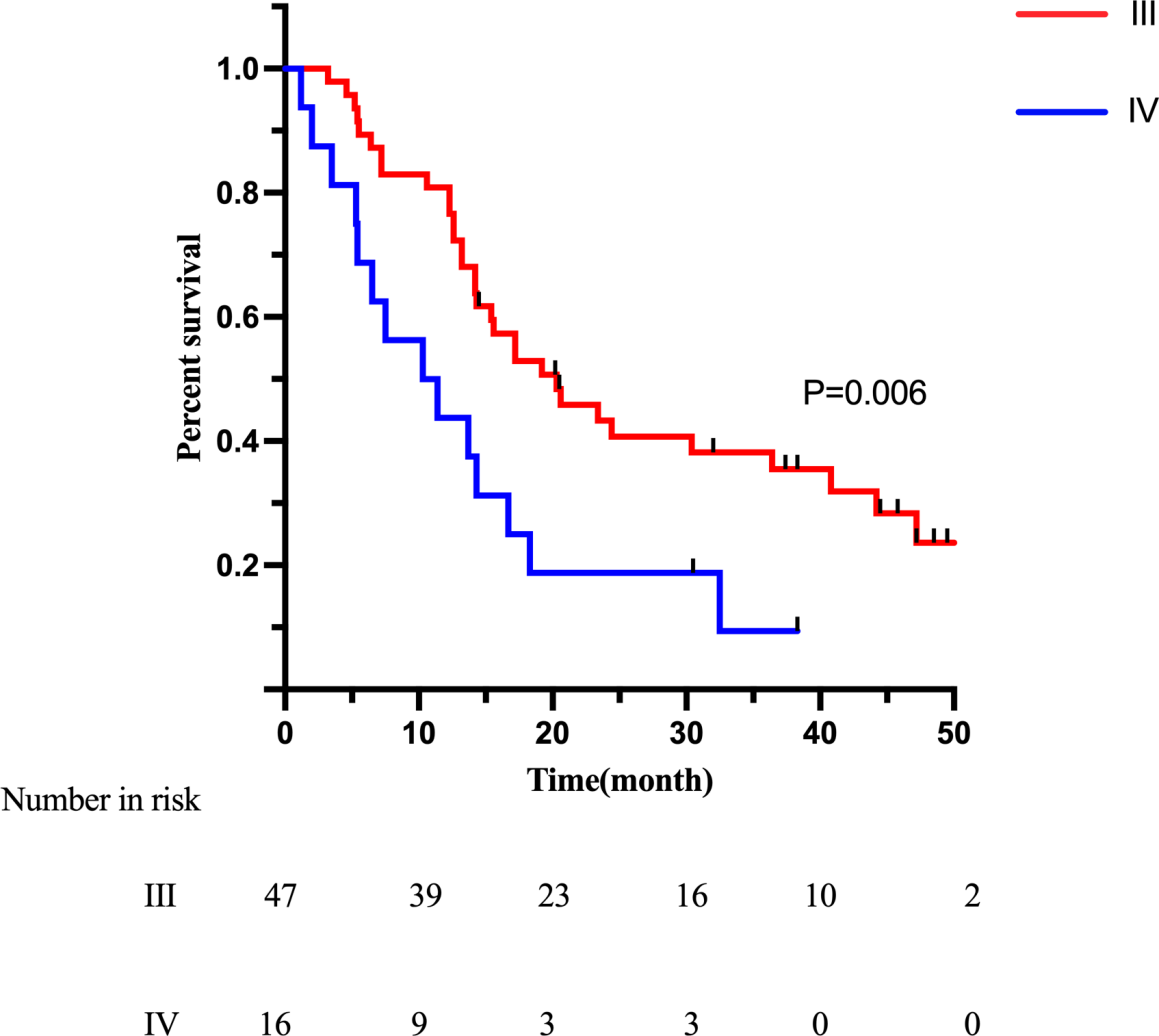
Kaplan-Meier analysis of overall survival stratified by Borrmann classification. GOOS.

**Figure 3:**
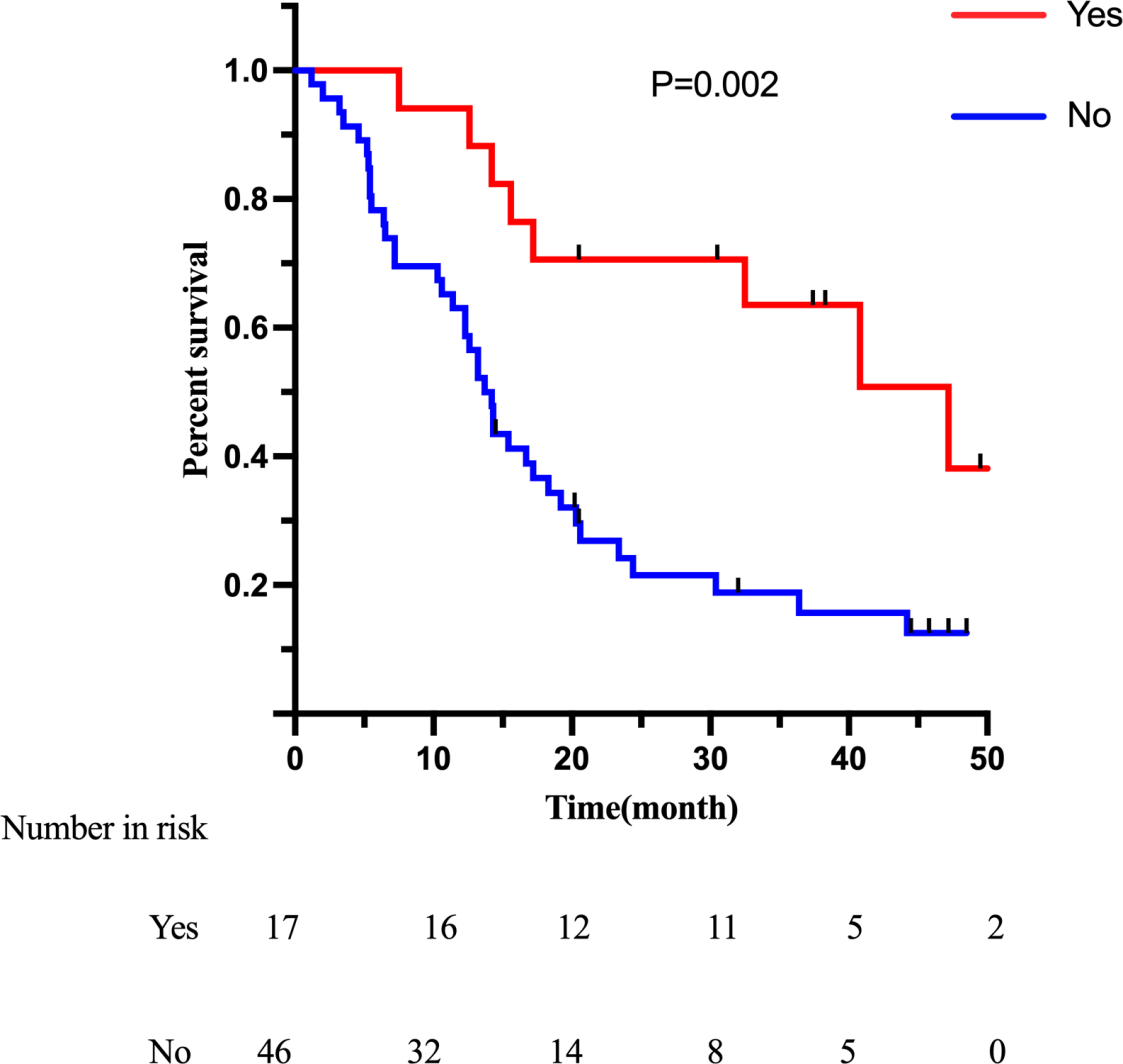
Kaplan-Meier analysis of overall survival stratified by conversion surgery status.

In survival analysis, the median survival time (MST) was 18.3 months in the LGJ group and 14.3 months in the NIT group (p = 0.453), with no statistically significant difference (**Figure 1**). Univariate Cox regression analysis showed that Borrmann classification (p < 0.001) and whether or not conversion surgery was performed (p < 0.001) were factors influencing survival (**Table 5**). Specifically, Borrmann type IV and failure to undergo conversion surgery were identified as potential risk factors. Multivariate analysis confirmed that Borrmann classification (p < 0.001) and the status of conversion surgery (p < 0.001) were independent prognostic factors for overall survival (OS). Given that conversion surgery and Borrmann classification were identified as independent prognostic factors, additional Kaplan-Meier analyses were performed stratified by these two variables. Patients with Borrmann type IV tumors had significantly poorer OS than those with III tumors (**Figure 2**). And patients who underwent conversion surgery showed significantly longer OS than those who did not undergo conversion surgery (**Figure 3**). These findings support the prognostic importance of tumor biology and successful conversion to resection in this population.

**Table 5:** Prognostic factors for overall survival.

Variables	Hazard ratio	95% CI	*P value*
Univariate Analysis			
Age (<65 or ≥65)	1.139	0.633-2.051	0.664
Sex (Male/Female)	1.293	0.706-2.369	0.405
BMI (<15 or ≥15)	0.545	0.278-1.413	0.260
ECOG (0/1 or 2)	0.885	0.454-1.724	0.719
QOL (<6 or ≥6)	1.003	0.552-1.823	0.991
Bormann classification (Type III or IV)	4.872	2.452-9.683	< 0.001
Conversion surgery (Yes/No)	0.71	0.021-0.239	< 0.001
CEA (<5 or ≥5)	1.764	0.985-3.159	0.056
PNI (<45 or ≥45)	0.765	0.418-1.401	0.386
PLR (<162 or ≥162)	1.332	0.728-2.439	0.353
NLR (<2.5 or ≥2.5)	0.778	0.428-1.415	0.411
Time to chemotherapy initiation	1.375	0.780-2.146	0.682
Multivariate Analysis			
Bormann classification (Type III or IV)	3.515	1.737-7.112	< 0.001
Conversion surgery (Yes/No)	0.074	0.021-0.256	< 0.001
CEA (<5 or ≥5)	1.670	0.916-3.043	0.094

PNI: Prognostic Nutritional Index, BMI: Body mass index, PLR: Platelet to lymphocyte ratio, NLR: Neutrophil to lymphocyte ratio.

It is worth noting that the variables included in the regression model, such as nutritional status, quality of life, inflammatory status, and tumor marker positivity, were all derived from baseline data.

## 4 Discussion

The treatment of advanced gastric cancer with gastric outlet obstruction (GOO) involves two main aspects: 1) early and effective relief or alleviation of obstructive symptoms, and 2) appropriate antitumor therapy. This retrospective study compared the short-term outcomes (postoperative complications, nutritional indicators, inflammatory and tumor markers) and long-term outcomes (overall survival, OS) of laparoscopic gastrojejunostomy (LGJ) versus nasointestinal tube (NIT) placement combined with chemotherapy in patients with incurable advanced GOO gastric cancer. Cox regression analysis was conducted to explore factors associated with OS.

To date, many studies have compared the short-term clinical efficacy of LGJ versus other palliative methods (9, 18–20). Our findings demonstrate that the incidence of severe postoperative complications after LGJ is extremely low, consistent with previous reports (21), indicating that LGJ is a safe and feasible approach for managing incurable GOO in advanced gastric cancer. In terms of nutritional status, GOOSS score, and quality of life, the LGJ group significantly outperformed the NIT group. Although direct comparisons between LGJ and NIT are scarce, our findings are generally in line with previous comparisons between LGJ and endoscopic stenting (22–24). Despite patients in the NIT group not achieving full oral intake, improvements in GOOSS and quality of life scores were still observed, likely due to: 1) improved nutrition and reduced gastric wall edema, 2) partial tumor regression, and 3) support and decompression effects of the feeding tube. It should be acknowledged that improvement in GOOSS or the ability to resume oral intake does not necessarily indicate that caloric and protein requirements were fully achieved. Because detailed daily nutritional intake data were not consistently recorded in this retrospective cohort, we could not directly compare actual caloric delivery between the two groups. This limitation may partly explain why some nutritional markers did not show significant differences despite better oral intake and QOL in the LGJ group. Future prospective studies should incorporate standardized dietician-led nutritional protocols and quantitative caloric/protein intake monitoring.

The NIT group had the advantages of simpler endoscopic procedures and minimal postoperative recovery, resulting in shorter hospital stays and earlier initiation of chemotherapy compared to the LGJ group. Because timely initiation of systemic therapy is critical in conversion treatment strategies, this delay could influence oncologic outcomes. Although adding this variable to the multivariate Cox model did not reveal an independent survival effect, the potential for delayed chemotherapy to offset some clinical benefits of LGJ warrants consideration. The nutritional and symptomatic improvements associated with LGJ must therefore be balanced against the need for expedient systemic therapy, particularly in patients for whom tumor biology necessitates early chemotherapy exposure.

Current research indicates that advanced malignancies are often systemic diseases. Increasing evidence suggests that elevated inflammatory markers (25, 26) may be associated with aggressive tumor biology and poor prognosis, whereas lower inflammatory states may predict better outcomes (27, 28). In this study, we analyzed baseline and post-treatment levels of PNI, NLR, and PLR in both groups. Compared to the LGJ group, the NIT group had a higher proportion of NLR-positive patients, while PNI and PLR showed no significant difference. This may be attributed to prolonged stimulation from the feeding tube on the tumor and mucosa, as well as hematologic disturbances caused by chemotherapy, suggesting that NLR findings should be interpreted with caution.

The CEA positivity rate was higher in the NIT group than in the LGJ group, which often indicates tumor progression or poor prognosis. However, no significant difference in overall survival was found between the groups. This inconsistency may be related to clinical heterogeneity between patients and the relatively small sample size of the study, which could introduce statistical bias.

Although patients in the LGJ group showed better nutritional status, GOOSS scores, and quality of life, the two groups did not differ significantly in survival or conversion success rates. This highlights the critical role of effective chemotherapy in determining overall survival for these patients. Multivariate analysis identified successful conversion surgery as an independent prognostic factor. The extremely low hazard ratio for conversion surgery (HR = 0.074) may reflect model overfitting given the small sample size, even though proportional hazard assumptions were met; this limitation should be considered when interpreting the results. In addition, Borrmann type IV was also an independent risk factor, as it is characterized by diffuse infiltration and poor response to chemotherapy, further emphasizing the importance of effective chemotherapy in these patients. Borrmann type IV was analyzed separately because of its diffuse growth pattern, frequent peritoneal dissemination, and poorer response to treatment (5).While LGJ was associated with greater improvements in GOOSS scores, nutritional parameters, and patient-reported quality of life, these advantages did not translate into a statistically significant survival difference compared with the NIT group. The symptomatic and nutritional benefits observed with LGJ therefore appear to reflect its ability to restore gastrointestinal continuity rather than an oncologic effect. Survival outcomes between the two approaches were broadly comparable, underscoring that the choice of intervention should be individualized based on symptom burden, nutritional needs, and overall clinical context rather than expectations of improved prognosis.

Therefore, compared to current standard palliative treatment options, LGJ may be a viable option for patients with good general condition. NIT combined with conversion therapy may allow some patients to avoid palliative surgery or stent-related complications and directly proceed to curative resection after effective conversion. However, further research is needed to identify which patients are more likely to benefit from conversion and what factors play decisive roles. For patients with poor cardiopulmonary function or high ECOG scores who may not tolerate surgery or anesthesia, NIT could help improve nutritional status and provide a window for palliative chemotherapy, thus potentially prolonging survival and representing an optimized choice.

This study has several limitations. First, although treatment choice was influenced by patient and family preference after counseling, the baseline characteristics between the LGJ and NIT groups were well balanced, suggesting that the magnitude of selection bias was limited. Because of this comparability and the relatively small sample size, additional methods such as propensity score matching were not applied, as they could further reduce statistical power. Nevertheless, the possibility of residual bias cannot be fully excluded and should be considered when interpreting the results. We plan to expand the cohort and collect more comprehensive variables in the future. Second, we did not assess outcomes in patients who received fewer than two chemotherapy cycles or who refused treatment. Moreover, the follow-up period was not long enough to determine 5-year survival, which requires further observation. The conversion protocol in this study was SOX-based chemotherapy, and as targeted and immunotherapies are increasingly applied clinically, the combination of NIT with targeted (29) or immunotherapies (30, 31) warrants further investigation.

## 5 Conclusion

Both LGJ and NIT are safe and feasible options for relieving GOO in patients with incurable advanced gastric cancer. Although there were no statistically significant differences in overall survival or conversion success rates, LGJ offers meaningful symptomatic relief and nutritional improvement for patients with malignant gastric outlet obstruction. These findings suggest that the primary advantages of LGJ lie in functional and quality-of-life benefits rather than survival extension, and treatment selection should prioritize patient-centered goals and clinical suitability.

## Data Availability

The raw data supporting the conclusions of this article will be made available by the authors, without undue reservation.

## References

[1] Sung H Ferlay J Siegel RL Laversanne M Soerjomataram I Jemal A . Global cancer statistics 2020: GLOBOCAN estimates of incidence and mortality worldwide for 36 cancers in 185 countries. CA Cancer J Clin. (2021) 71:209–49. doi: 10.3322/caac.21660. PMID: 33538338

[2] Wang Z Han W Xue F Zhao Y Wu P Chen Y . Nationwide gastric cancer prevention in China, 2021-2035: a decision analysis on effect, affordability and cost-effectiveness optimisation. Gut. (2022) 71:2391–400. doi: 10.1136/gutjnl-2021-325948. PMID: 35902213

[3] Chia NY Tan P . Molecular classification of gastric cancer. Ann Oncol. (2016) 27:763–9. doi: 10.1093/annonc/mdw040. PMID: 26861606

[4] Guan WL He Y Xu RH . Gastric cancer treatment: recent progress and future perspectives. J Hematol Oncol. (2023) 16:57. doi: 10.1186/s13045-023-01451-3. PMID: 37245017 PMC10225110

[5] Smyth EC Nilsson M Grabsch HI van Grieken NCT Lordick F . Gastric cancer. Lancet. (2020) 396:635–48. doi: 10.1016/b978-0-443-15609-0.00017-9. PMID: 32861308

[6] Joshi SS Badgwell BD . Current treatment and recent progress in gastric cancer. CA Cancer J Clin. (2021) 71:264–79. doi: 10.3322/caac.21657. PMID: 33592120 PMC9927927

[7] Potz BA Miner TJ . Surgical palliation of gastric outlet obstruction in advanced Malignancy. World J Gastrointest Surg. (2016) 8:545–55. doi: 10.4240/wjgs.v8.i8.545. PMID: 27648158 PMC5003933

[8] Koop AH Palmer WC Stancampiano FF . Gastric outlet obstruction: A red flag, potentially manageable. Cleve Clin J Med. (2019) 86:345–53. doi: 10.3949/ccjm.86a.18035. PMID: 31066665

[9] Troncone E Fugazza A Cappello A Del Vecchio Blanco G Monteleone G Repici A . Malignant gastric outlet obstruction: Which is the best therapeutic option? World J Gastroenterol. (2020) 26:1847–60. doi: 10.3748/wjg.v26.i16.1847. PMID: 32390697 PMC7201143

[10] Manuel-Vázquez A Latorre-Fragua R Ramiro-Pérez C López-Marcano A De la Plaza-Llamas R Ramia JM . Laparoscopic gastrojejunostomy for gastric outlet obstruction in patients with unresectable hepatopancreatobiliary cancers: A personal series and systematic review of the literature. World J Gastroenterol. (2018) 24:1978–88. doi: 10.3748/wjg.v24.i18.1978. PMID: 29760541 PMC5949711

[11] Miyazaki Y Takiguchi S Takahashi T Kurokawa Y Makino T Yamasaki M . Treatment of gastric outlet obstruction that results from unresectable gastric cancer: Current evidence. World J Gastrointest Endosc. (2016) 8:165–72. doi: 10.4253/wjge.v8.i3.165. PMID: 26862366 PMC4734975

[12] Ahmed O Lee JH Thompson CC Faulx A . AGA clinical practice update on the optimal management of the Malignant alimentary tract obstruction: Expert review. Clin Gastroenterol Hepatol. (2021) 19:1780–8. doi: 10.1016/j.cgh.2021.03.046. PMID: 33813072

[13] Boškoski I Tringali A Familiari P Mutignani M Costamagna G . Self-expandable metallic stents for Malignant gastric outlet obstruction. Adv Ther. (2010) 27:691–703. doi: 10.1007/s12325-010-0061-2 20737260

[14] Wang C Zhang X Lin S Yang C Zhou B Mi Y . Superiority of laparoscopic gastrojejunostomy combined with multimodality therapy for gastric outlet obstruction caused by advanced gastric cancer. Front Oncol. (2023) 12:814283:1145579. doi: 10.3389/fonc.2022.814283. PMID: 35155250 PMC8832489

[15] Milsom SA Sweeting JA Sheahan H Haemmerle E Windsor JA . Naso-enteric tube placement: A review of methods to confirm tip location, global applicability and requirements. World J Surg. (2015) 39:2243–52. doi: 10.1007/s00268-015-3077-6. PMID: 25900711

[16] Ley D Austin K Wilson KA Saha S . Tutorial on adult enteral tube feeding: Indications, placement, removal, complications, and ethics. Jpen J Parenter Enteral Nutr. (2023) 47:677–85. doi: 10.1002/jpen.2510. PMID: 37122159

[17] Karpeh MS . Palliative treatment and the role of surgical resection in gastric cancer. Dig Surg. (2013) 30:174–80. doi: 10.1159/000351177. PMID: 23867595

[18] Tsukada K . Metallic stent placement or gastroenterostomy for gastric outlet obstruction caused by gastric cancer? J Gastroenterol. (2005) 40:1007–8. doi: 10.1007/s00535-005-1700-2. PMID: 16261444

[19] Jeurnink SM van Eijck CHJ Steyerberg EW Kuipers EJ Siersema PD . Stent versus gastrojejunostomy for the palliation of gastric outlet obstruction: a systematic review. BMC Gastroenterol. (2007) 7:18. doi: 10.1186/1471-230x-7-18. PMID: 17559659 PMC1904222

[20] Yoshida K Yamaguchi K Okumura N Tanahashi T Kodera Y . Is conversion therapy possible in stage IV gastric cancer: the proposal of new biological categories of classification. Gastric Cancer. (2016) 19:329–38. doi: 10.1007/s10120-015-0575-z. PMID: 26643880 PMC4824831

[21] Arigami T Uenosono Y Ishigami S Yanagita S Okubo K Uchikado Y . Clinical impact of stomach-partitioning gastrojejunostomy with Braun enteroenterostomy for patients with gastric outlet obstruction caused by unresectable gastric cancer. Anticancer Res. (2016) 36:5431–6. doi: 10.21873/anticanres.11121. PMID: 27798911

[22] Upchurch E Ragusa M Cirocchi R . Stent placement versus surgical palliation for adults with Malignant gastric outlet obstruction. Cochrane Database Syst Rev. (2018) 5:CD012506. doi: 10.1002/14651858.cd012506.pub2. PMID: 29845610 PMC6494580

[23] Jang S Stevens T Lopez R Bhatt A Vargo JJ . Superiority of gastrojejunostomy over endoscopic stenting for palliation of Malignant gastric outlet obstruction. Clin Gastroenterol Hepatol. (2019) 17:1295–302 e1. doi: 10.1016/j.cgh.2018.10.042. PMID: 30391433

[24] Ojima T Nakamori M Nakamura M Katsuda M Hayata K Yamaue H . Laparoscopic gastrojejunostomy for patients with unresectable gastric cancer with gastric outlet obstruction. J Gastrointest Surg. (2017) 21:1220–5. doi: 10.1007/s11605-017-3387-0. PMID: 28224464

[25] Diakos CI Charles KA McMillan DC Clarke SJ . Cancer-related inflammation and treatment effectiveness. Lancet Oncol. (2014) 15:e493–503. doi: 10.1016/s1470-2045(14)70263-3. PMID: 25281468

[26] Shinko D Diakos CI Clarke SJ Charles KA . Cancer-related systemic inflammation: The challenges and therapeutic opportunities for personalized medicine. Clin Pharmacol Ther. (2017) 102:599–610. doi: 10.1002/cpt.789. PMID: 28699186

[27] Guo L Wang Q Chen K Liu HP Chen X . Prognostic value of combination of inflammatory and tumor markers in resectable gastric cancer. J Gastrointest Surg. (2021) 25:2470–83. doi: 10.1007/s11605-021-04944-z. PMID: 33575904

[28] Powell AGMT Parkinson D Patel N Chan D Christian A Lewis WG . Prognostic significance of serum inflammatory markers in gastric cancer. J Gastrointest Surg. (2018) 22:595–605. doi: 10.26226/morressier.59a6b348d462b80290b551fd 29234999 PMC5869874

[29] Meric-Bernstam F Johnson AM Dumbrava EEI Raghav K Balaji K Bhatt M . Advances in HER2-targeted therapy: Novel agents and opportunities beyond breast and gastric cancer. Clin Cancer Res. (2019) 25:2033–41. doi: 10.1158/1078-0432.ccr-18-2275. PMID: 30442682 PMC6445731

[30] Hogner A Moehler M . Immunotherapy in gastric cancer. Curr Oncol. (2022) 29:1559–74. doi: 10.3390/curroncol29030131 PMC8946975 35323331

[31] Li K Zhang A Li X Zhang H Zhao L . Advances in clinical immunotherapy for gastric cancer. Biochim Biophys Acta Rev Cancer. (2021) 1876:188615. doi: 10.1016/j.bbcan.2021.188615. PMID: 34403771

